# Ficolin-2 inhibitors are present in sera after prolonged storage at −80 °C

**DOI:** 10.7717/peerj.2705

**Published:** 2016-11-17

**Authors:** Kimball Aaron Geno, Richard E. Kennedy, Patricia Sawyer, Cynthia J. Brown, Moon H. Nahm

**Affiliations:** 1 Division of Pulmonary, Allergy, and Critical Care Medicine, University of Alabama at Birmingham, Birmingham, AL, United States; 2 Division of Gerontology, Geriatrics, and Palliative Care, University of Alabama at Birmingham, Birmingham, AL, United States; 3 Comprehensive Center for Healthy Aging, University of Alabama at Birmingham, Birmingham, AL, United States; 4 Birmingham/Atlanta Geriatric Research, Education, and Clinical Center, Birmingham Veteran’s Affairs Medical Center, Birmingham, AL, United States; 5 Department of Microbiology, University of Alabama at Birmingham, Birmingham, AL, United States

**Keywords:** Ficolins, Lectins, Storage artifacts, Lectin pathway, Complement

## Abstract

Ficolins can activate the lectin pathway of the complement system that provides innate immune protection against pathogens, marks host cellular debris for clearance, and promotes inflammation. Baseline inflammation increases with aging in a phenomenon known as “inflammaging.” Although IL-6 and C-reactive protein are known to increase with age, contributions of many complement factors, including ficolins, to inflammaging have been little studied.

Ficolin-2 is abundant in human serum and can recognize many target structures; therefore, ficolin-2 has potential to contribute to inflammaging. We hypothesized that inflammaging would alter ficolin-2 levels among older adults and examined 360 archived sera collected from older individuals. We found that these sera had apparently reduced ficolin-2 levels and that 84.2% of archived sera exhibited ficolin-2 inhibitors, which suppressed apparent amounts of ficolin-2 detected by enzyme-linked immunosorbent assay. Fresh serum samples were obtained from donors whose archived sera showed inhibitors, but the fresh sera did not have ficolin-2 inhibitors. Ficolin-2 inhibitors were present in other long-stored sera from younger persons. Furthermore, noninhibiting samples and fresh sera from older adults had apparently normal amounts of ficolin-2. Thus, ficolin-2 inhibitors may arise as an artifact of long-term storage of serum at −80 °C.

## Introduction

The complement system is critical to both innate and adaptive immunity. Complement activation, reviewed in reference (Walport, 2001), leads to opsonization of foreign particles or dying host cells for clearance by phagocytosis or to lysis of susceptible targets through formation of the membrane attack complex (MAC). The complement system is also important in inflammation, as many complement fragments, especially C3a and C5a, are inflammatory. Complement activation occurs through three pathways, known as the alternative, classical, and lectin pathways. In the alternative pathway, C3 is spontaneously hydrolyzed to C3b, which deposits on nearby target surfaces and may activate downstream portions of the complement cascade, including the MAC, through interactions with alternative pathway-specific elements such as factor B. In the classical pathway, C1q recognizes antibodies bound to a target and activates serine proteases (C1r, C1s) to produce the C3 convertase, C4b2a, which causes further C3 cleavage and deposition of C3b onto the target surface resulting in further complement activation and MAC formation. The lectin pathway is similar to the classical pathway but is initiated when a specialized activator molecule recognizes a target structure and activates the mannose binding lectin(MBL)/ficolin-associated serine proteases (MASPs), which generate the classical pathway C3 convertase, C4b2a.

The lectin pathway is phylogenetically old and thus likely incorporated into many fundamental processes. Activators of this pathway include the collectins, with lectin domains (MBL, CL-K1, CL-L1, Keshi et al., 2006; Ohtani et al., 1999), and the ficolins, with fibrinogen-like domains (ficolin-1, -2, and -3, recently reviewed in reference (Endo, Matsushita & Fujita, 2011; Matsushita, 2013)). The fibrinogen-like domains of ficolins preferentially bind to acetyl groups (Matsushita, 2013), but ficolin-2 has a fibrinogen-like domain with three additional binding sites relative to the other human ficolins (Endo, Matsushita & Fujita, 2011), and consequently recognizes a larger number of target structures. These include non-acetylated molecular structures such as phosphocholine and heparin (Matsushita, 2013; Vassal-Stermann et al., 2014). Ficolin-2 binds to many bacteria (Matsushita, 2013), fungi (Bidula et al., 2015), and viruses (Liu et al., 2009; Pan et al., 2012). In addition, ficolin-2 binds to apoptotic cells and mitochondria (Brinkmann et al., 2013; Endo, Matsushita & Fujita, 2011), suggesting a role in host homeostasis. Perhaps the most well-characterized interaction of ficolin-2 is its binding to pneumococcal serotype 11A (Brady et al., 2014a; Krarup et al., 2005), which micro-evolves *in vivo* into serotype 11E to escape ficolin-2-mediated immunity (Brady et al., 2014a; Calix & Nahm, 2010). Reflecting ficolin-2-mediated innate immunity to pneumococcal serotype 11A, invasive disease by this serotype is very rare among children (Brady et al., 2014a; Pilishvili et al., 2010).

Complement may also be involved in the aging of immune function. A prominent feature of immunity in aging is an increased baseline of inflammation, with increased levels of IL-6 and C-reactive protein (CRP). This increase is often termed “inflammaging” and has been associated with the age-associated decline in immune function (Franceschi et al., 2000). Complement activity is stated to be increased with aging, but little direct evidence is available. The few studies examining complement levels in older adults have offered no clear conclusions, as results have often been contradictory (Simell et al., 2011). Given the wide binding array of ficolin-2 and its potential roles in removing host cellular debris (Endo, Matsushita & Fujita, 2011), ficolin-2 may be important in inflammaging. Ficolin-2 levels are shown to increase during early childhood, reaching maximal levels between the ages 1 and 4 years before slightly declining in adulthood (Sallenbach et al., 2011); however, neither levels of ficolin-2 nor its function has been examined in older adults. We hypothesized that ficolin-2 levels or activity would be altered among older adults. To investigate this hypothesis, we studied ficolin-2 levels and activity with a collection of archived sera obtained from older adults during the University of Alabama at Birmingham (UAB) Study of Aging (Allman, Sawyer & Roseman, 2006; Salanitro et al., 2012).

## Materials and Methods

### Sera

The collection of sera for the UAB Study of Aging, whose participants were at least 69 years old at the time of blood draw, has been previously described (Allman, Sawyer & Roseman, 2006; Salanitro et al., 2012). IRB approval (protocol X140618001) was obtained for the use of archived samples from the UAB Study of Aging and the collection of fresh samples from UAB Study of Aging participants, which were collected in glass Vacutainer^®^serum collection tubes (BD 366441) with written consent from participants. Normal human sera (NHS) were obtained from healthy young adult volunteers in glass and plastic (BD 367820) Vacutainer^®^collection tubes under an IRB-approved protocol (protocol X120719005) with written consent from the volunteers.

### Ficolin-2 quantitation

Ficolin-2 levels were determined using a commercial ELISA (HyCult HK336-02). Values for young, healthy controls were previously reported (Brady et al., 2014c). In mixing experiments, each serum sample was tested alone at 20-fold dilution (15 µl serum + 285 µl kit dilution buffer). For mixed samples, 15 µl of each sample was mixed with 270 µl kit dilution buffer, with the result that the expected value represents the sum of the individual samples.

### Ficolin-2 inhibition assay

Inhibition assays were performed as previously-described (Brady et al., 2014a; Geno, Spencer & Nahm, 2015) with modifications. Briefly, test sera were diluted to 20% in gelatin veronal buffer (GVB; 142 mM NaCl, 0.15 mM CaCl_2_, 0.5 mM MgCl_2_, 0.1% gelatin, 5 mM sodium barbital, 0.004% NaN_3_, pH = 7.4) and heat-inactivated at 56 °C for 45 min to remove endogenous ficolin-2 activity. Serotype 11A frozen bacterial stocks, prepared as previously described (Brady et al., 2014a), were thawed, washed, and resuspended to 10^6^ cfu/ml in GVB. Twenty-five microliters of serum were placed in the wells of V-bottom 96-well plates (Nunc), and 25 µl of ficolin-2-containing cell culture supernatant was added to each well except for relevant controls. Fifty microliters of bacterial solution were added to each well, and the plate was shaken at 37 °C at 700 rpm on a Bellco Biotechnology mini-orbital shaker. Bacteria were washed, and deposited ficolin-2 was detected using a ficolin-2-specific antibody (Pierce ABS 005-19-02, 1:1,000 dilution) with a phycoerythrin-conjugated secondary antibody (Southern Biotech 1010-09, 1:2,500 dilution) and flow cytometry as previously described (Brady et al., 2014a; Geno, Spencer & Nahm, 2015).

### Ficolin-2 immunoblotting

Serum samples (3 µl per lane) were assayed for ficolin-2 by SDS-12%PAGE as previously described (Brady et al., 2014b).

### Far-western blotting

Ficolin-2 was purified from supernatants of the CHO cell derived cell line huf2E as previously described (Geno, Spencer & Nahm, 2015) and biotinylated using a commercial kit (Thermo 21425). Two microliters of serum were mixed with 18 µl H_2_O and 20 µl 2X SDS-PAGE loading buffer (Bio-Rad, supplemented to 5% v/v 2-mercapto ethanol) and analyzed by SDS-8%PAGE. Gel contents were transferred to nitrocellulose by semi-dry blotting, blocked in 5% powdered skim milk, and probed with 2.5 µg biotinylated ficolin-2 in 10 ml tris-buffered saline-Tween-20 (TBST; 50 mM Tris, 100 mM NaCl, pH 7.4 supplemented with 0.05% Tween-20) at 4 °C overnight. After 3 10-ml washes in TBST, membranes were probed with IRDye^®^800CW streptavidin (Li-Cor) diluted 1:20,000 in 10 ml TBST + 0.05% SDS for 2 h at room temperature. After six 10-ml washes in TBST, blots were visualized by a Li-Cor Odyssey infrared imager.

### Statistics

UAB Study of Aging participant records were stratified by their ficolin-2 inhibition status, and selected parameters were compared between groups by Wilcoxon rank-sum test for continuous variables or chi-squared test for categorical variables. Elsewhere, for single comparisons, data were analyzed using *t*-tests assuming unequal variances, and for multiple comparisons, data were analyzed using one-way analysis of variance (ANOVA) with Tukey’s post-hoc test, unless otherwise specified.

## Results

### Many archived sera have ficolin-2 inhibitors

**Figure 1:**
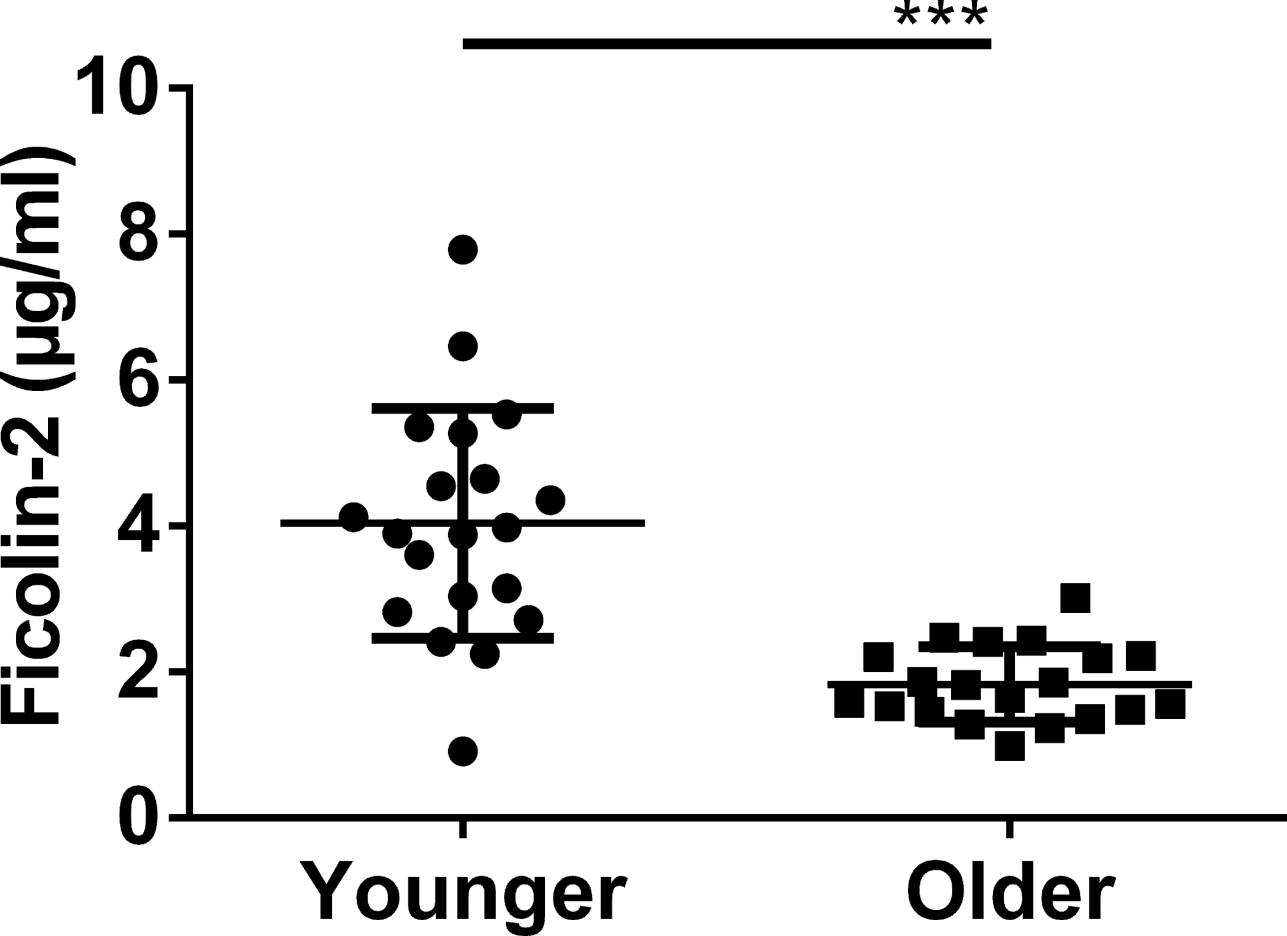
Archived older adults’ sera are significantly reduced in ficolin-2 content relative to healthy young controls. Twenty sera were selected at random from UAB Study of Aging archived sera and assayed for ficolin-2 by a commercial ELISA (“Older”). Each data point is shown along with mean and standard deviation. ***, *P* < 0.001 by unpaired *t*-test assuming unequal variances. Values for young, healthy controls (“Younger”) were previously reported by our laboratory (Brady et al., 2014a) and used here with permission.

We hypothesized that ficolin-2 would be elevated among older adults. To directly test this hypothesis, we determined ficolin-2 levels of 20 sera randomly selected from 360 archived serum samples collected from older adults (age ≥ 69 years at the time of blood draw) during the UAB Study of Aging (Allman, Sawyer & Roseman, 2006; Salanitro et al., 2012). Contrary to our expectations, as shown in Fig. 1, the older adults’ sera exhibited nearly 2-fold less ficolin-2 than a panel of 20 healthy young adult donors, whose ficolin-2 levels we reported previously (Brady et al., 2014c).

**Figure 2:**
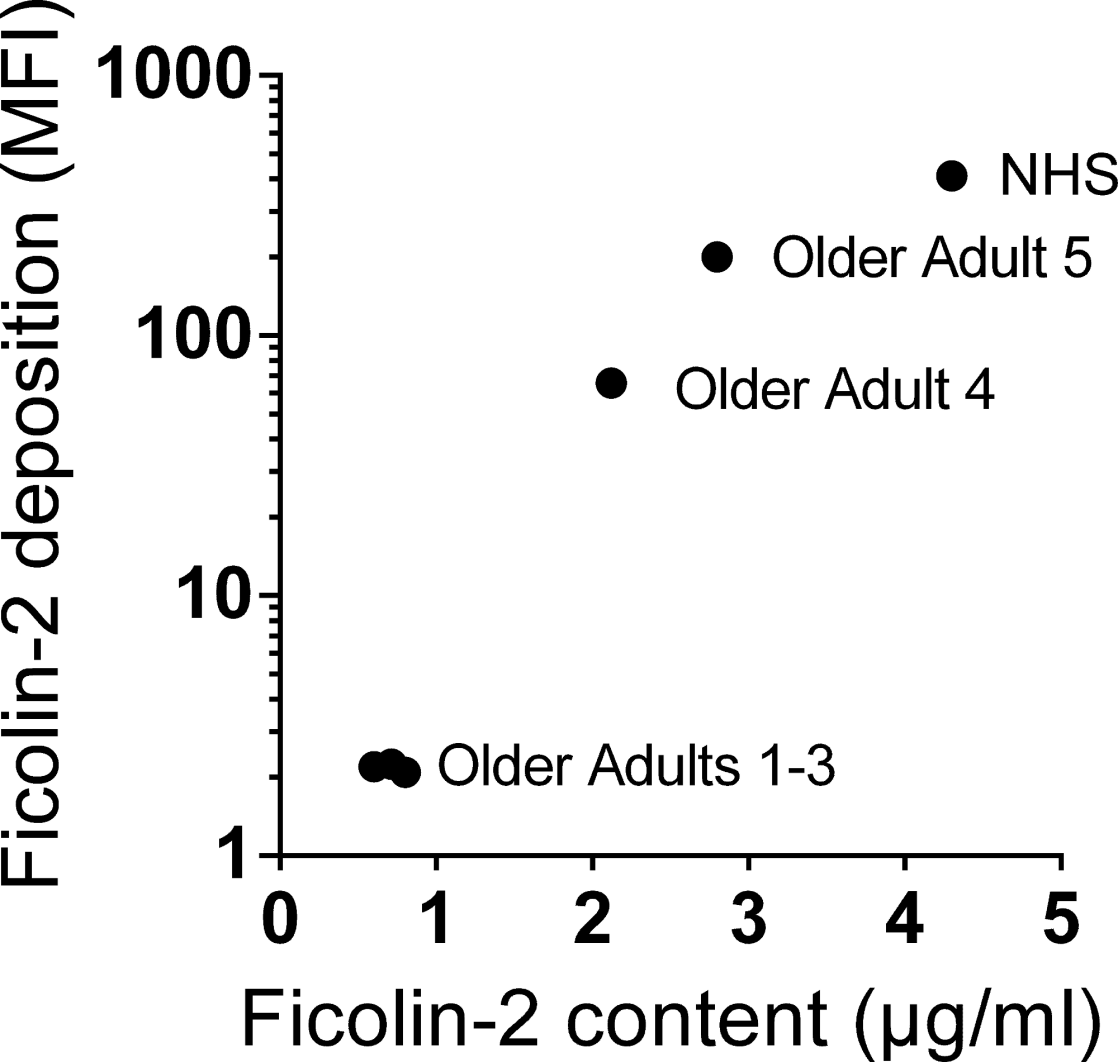
Some older individuals’ sera deposit disproportionately little ficolin-2 on serotype 11A bacteria. Sera were evaluated for ficolin-2 deposition on serotype 11A pneumococcus by flow cytometry and for ficolin-2 content by ELISA. NHS, normal human serum collected from a healthy young adult volunteer.

**Figure 3:**
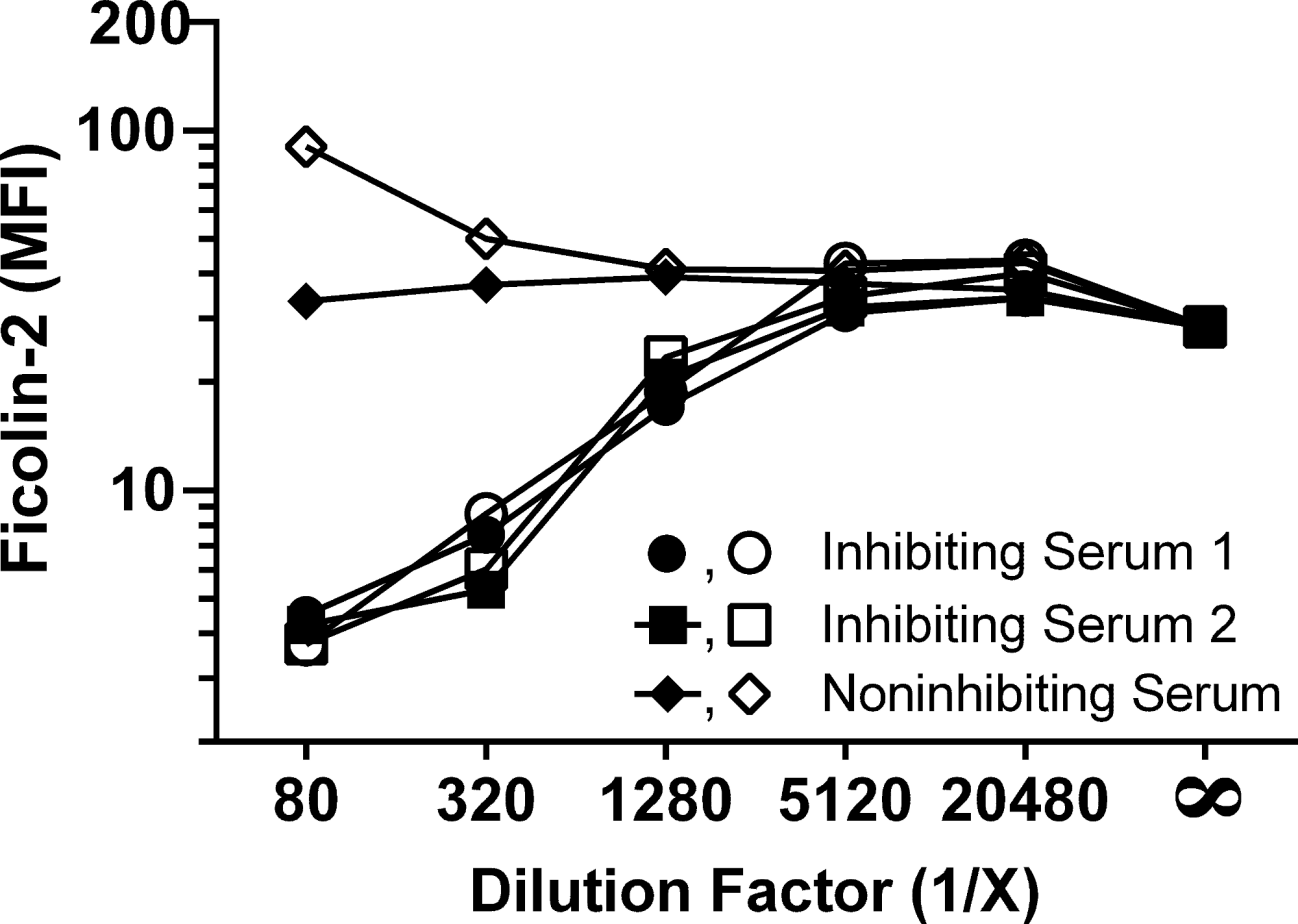
Some older adults’ sera have heat-resistant ficolin-2 inhibition. Sera were inactivated at 56 °C (solid symbols) or held at room temperature (open symbols) for 45 minutes prior to serial dilution, mixing with recombinant ficolin-2, and incubation with serotype 11A pneumococci. The elevated signal at lower dilutions of the noninhibiting serum sample without heat inactivation is accounted for by the innate ficolin-2 within this sample.

To confirm this unexpectedly low level of ficolin-2 in older adults’ sera, we initially examined five sera from older adults for ficolin-2 deposition on pneumococcal serotype 11A, which we and others have previously shown is bound by ficolin-2 (Brady et al., 2014a; Krarup et al., 2005). Three sera failed to deposit appreciable amounts of ficolin-2 on serotype 11A bacteria, while the other two exhibited moderately reduced deposition relative to our NHS control (Fig. 2, *Y* axis). However, we noted that three of the older adults’ sera had disproportionately low ficolin-2 binding to serotype 11A compared to their ficolin-2 levels (Fig. 2, *X* axis), and we hypothesized that the sera contained ficolin-2 inhibitors. When we examined these sera for inhibition of ficolin-2 binding to serotype 11A pneumococci, the three without appreciable ficolin-2 deposition had inhibitors that were of high-titer and heat-resistant (representative data shown in Fig. 3). When we tested all 360 sera at 20-fold dilution, 303 (84.2%) inhibited ficolin-2.

### Ficolin-2 inhibitors interfere with ficolin-2 quantitation by ELISA

**Figure 4:**
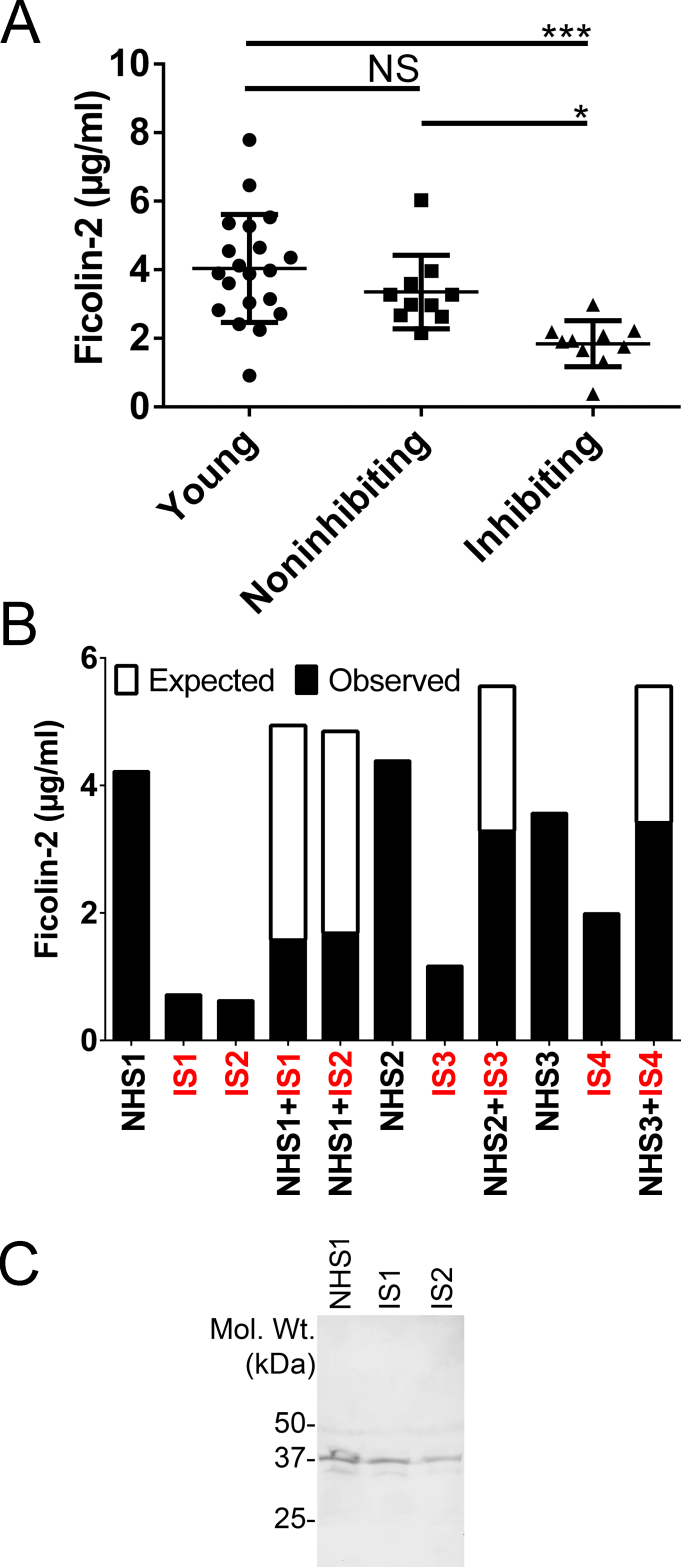
Inhibitors in archived sera interfere with an ELISA to quantify ficolin-2. (A) Ten inhibiting sera and ten noninhibiting sera were selected at random from UAB Study of Aging archived sera and assayed for ficolin-2 by a commercial ELISA. Each data point is shown along with mean and standard deviation. NS, not significant; *, *P* < 0.05; ***, *P* < 0.001 by one-way ANOVA with Tukey’s post-hoc test. Values for young, healthy controls were previously reported by our laboratory (Brady et al., 2014a). (B) Inhibitors in archived sera interfere with a commercial ficolin-2 ELISA. Three normal human sera (NHS1-3) and four inhibiting sera (IS1-4) were assayed by a commercial ELISA alone or in mixture. Mixtures were designed so that observed ficolin-2 would represent the sum of the samples (see *Materials and Methods*). Open bars represent the sum of the samples and thus the expected value of the assay. (C) Ficolin-2 immunoblot of sera tested in B suggests that IS1 and IS2 have comparable levels of ficolin-2 to NHS1. Ficolin-2 appears as a band of ∼37 kDa under these conditions.

We hypothesized that the inhibiting sera would have lower ficolin-2 levels by ELISA, and we randomly selected ten sera with and ten sera without inhibitors and assayed their ficolin-2 levels (Fig. 4A). The non-inhibiting sera had no significant difference in ficolin-2 content compared to our previously-reported values from young, healthy controls (Brady et al., 2014c), suggesting that the older adults with no inhibitors have normal levels of ficolin-2. In contrast, the inhibiting sera showed very low levels of ficolin-2.

Because we previously found that another ficolin-2 inhibitor can interfere with the ficolin-2 ELISA, (Brady et al., 2014c), we mixed three NHS controls independently with four inhibiting sera and measured ficolin-2 levels in the serum mixtures. Although the sera were mixed so that the ficolin-2 signal would increase, the resulting assay readout was in each case less than the NHS control alone, consistent with interference with the assay (Fig. 4B). By contrast, when we examined ficolin-2 levels of one NHS sample and two inhibiting samples by western blot, both inhibiting and non-inhibiting samples exhibited similar amounts of ficolin-2 (Fig. 4C). Thus, the inhibitor may not degrade endogenous ficolin-2 in the sera but interfere with the ELISA, likely competing with the ficolin-2-specific monoclonal antibody for ficolin-2 binding. It is also conceivable that ficolin-2 may become denatured during storage, which could additionally suppress apparent ficolin-2 levels.

### Presence of ficolin-2 inhibitors shows no association with inflammaging markers

Inflammaging is characterized by elevated levels of inflammatory cytokines such as IL-6 (Ershler, 1993) and acute phase reactants such as CRP (Woloshin & Schwartz, 2005) among older adults. To investigate biological significance of the inhibitors, we correlated inflammaging-associated markers among the two groups with and without inhibitors. IL-6 levels in both groups were significantly greater than a recently reported 95th percentile reference limit (4.45 pg/ml) in healthy adults younger than 65 years (*P* < 0.001 for inhibiting and *P* < 0.01 for noninhibiting perons in one-sample *t*-tests versus 4.45 pg/ml) (Todd et al., 2013). Nonetheless, as shown in Table 1, IL-6 and CRP levels were not significantly different between individuals with ficolin-2 inhibitors and individuals without. In addition, there was no significant difference observed in mean time to death between the two groups. Thus, presence of inhibitors did not suggest any biological differences.

**Table 1:** Comorbidities and inflammatory markers associated with inhibiting and noninhibiting sera.

	Inhibition	No inhibition
Number of samples	303	57
Time to death (years)	8.28 +/−0.07^a^	8.49 +/−0.14
IL-6 (pg/ml)	7.50 +/−15.88	7.42 +/−7.93
C-reactive protein (mg/L)	0.756 +/−1.27	0.922 +/−2.30
Asthma and COPD^b^ [n (%)]	34 (11.2)	7 (12.3)
Diabetes [n (%)]	66 (21.8)	11 (19.3)
Non-skin cancer [n (%)]	51 (16.8)	8 (14.0)
NSAID^c^ use [n (%)]	70 (23.1)^*^	6 (10.5)

**Notes.**

a Mean +/−standard deviation.

b Chronic obstructive pulmonary disorder.

c Non-steroidal anti-inflammatory drug.

* *P* < 0.05 by chi-square test.

### Fresh sera from UAB Study of Aging participants do not inhibit ficolin-2

**Figure 5:**
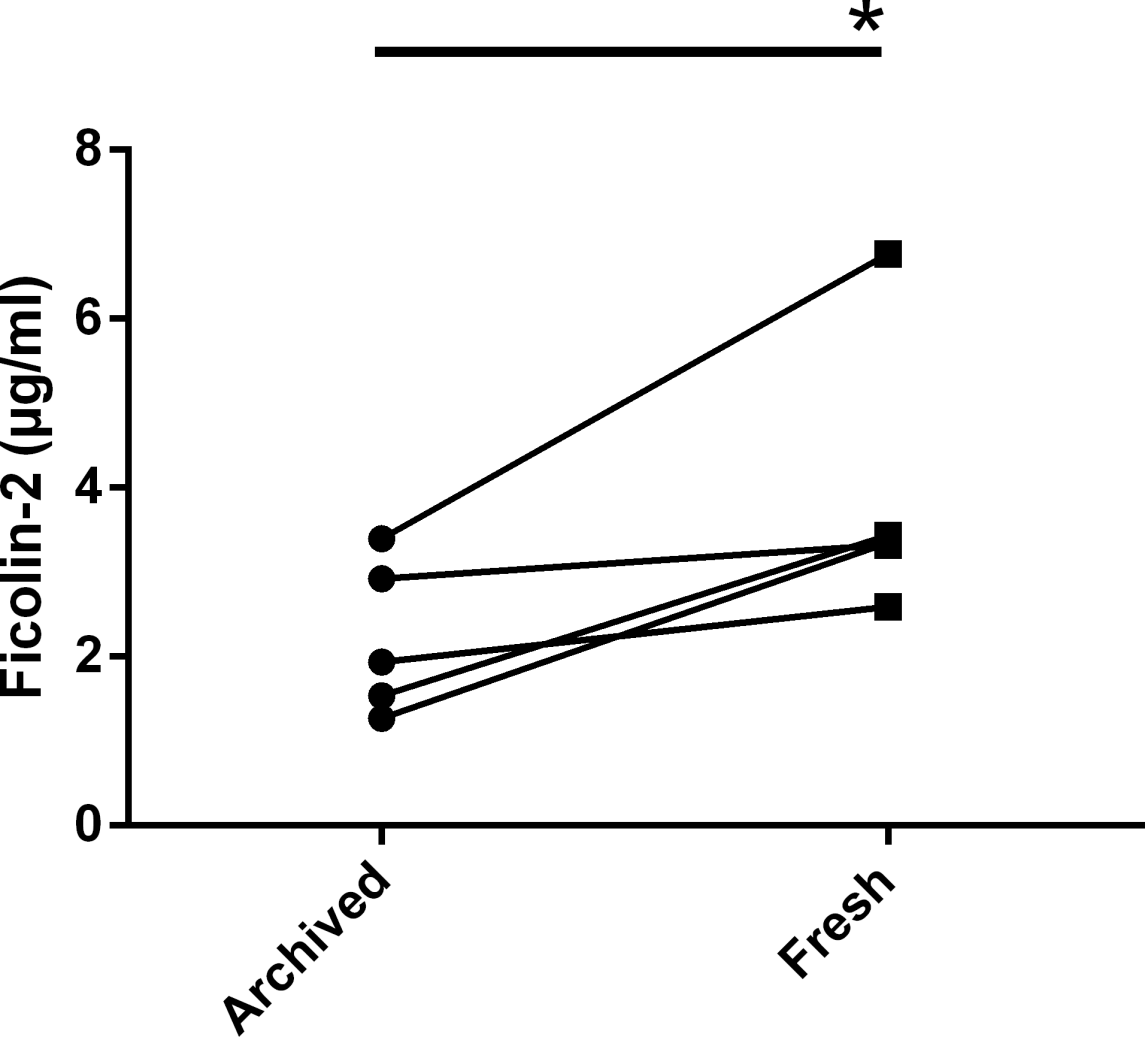
Freshly-collected sera from UAB Study of Aging participants whose archived samples inhibit ficolin-2 have significantly more ficolin-2 than their archived counterparts. Sera were assayed for ficolin-2 content by a commercial ELISA. *, *P* < 0.05 by paired *t*-test; connecting lines show matched samples. The mean ficolin-2 concentration (±SEM) was 2.21 ± 0.41 µg/ml for archived sera and 3.89 ± 0.73 µg/ml for matched freshly-collected sera.

Archived serum samples did not permit additional studies of inhibitors due to limitations in their volume. In order to study the inhibitors in detail, we obtained fresh sera from five original participants in the UAB Study of Aging whose archived samples inhibited ficolin-2. To our surprise, sera from these individuals failed to inhibit ficolin-2 and exhibited significantly greater ficolin-2 levels than their archived counterparts that were within the normal range of our healthy controls (Fig. 5). This finding raised the possibility that the inhibitors were artifacts of long-term freezer storage; therefore we examined ten random samples chosen from another set of sera stored long-term (∼12–18 years) in our laboratory for ficolin-2 inhibition. Two of ten sera (both collected in 1998) exhibited ficolin-2 inhibition, suggesting that the development of ficolin-2 inhibition during prolonged storage may be a broadly applicable phenomenon.

### Inhibiting samples exhibit molecules that bind ficolin-2

**Figure 6:**
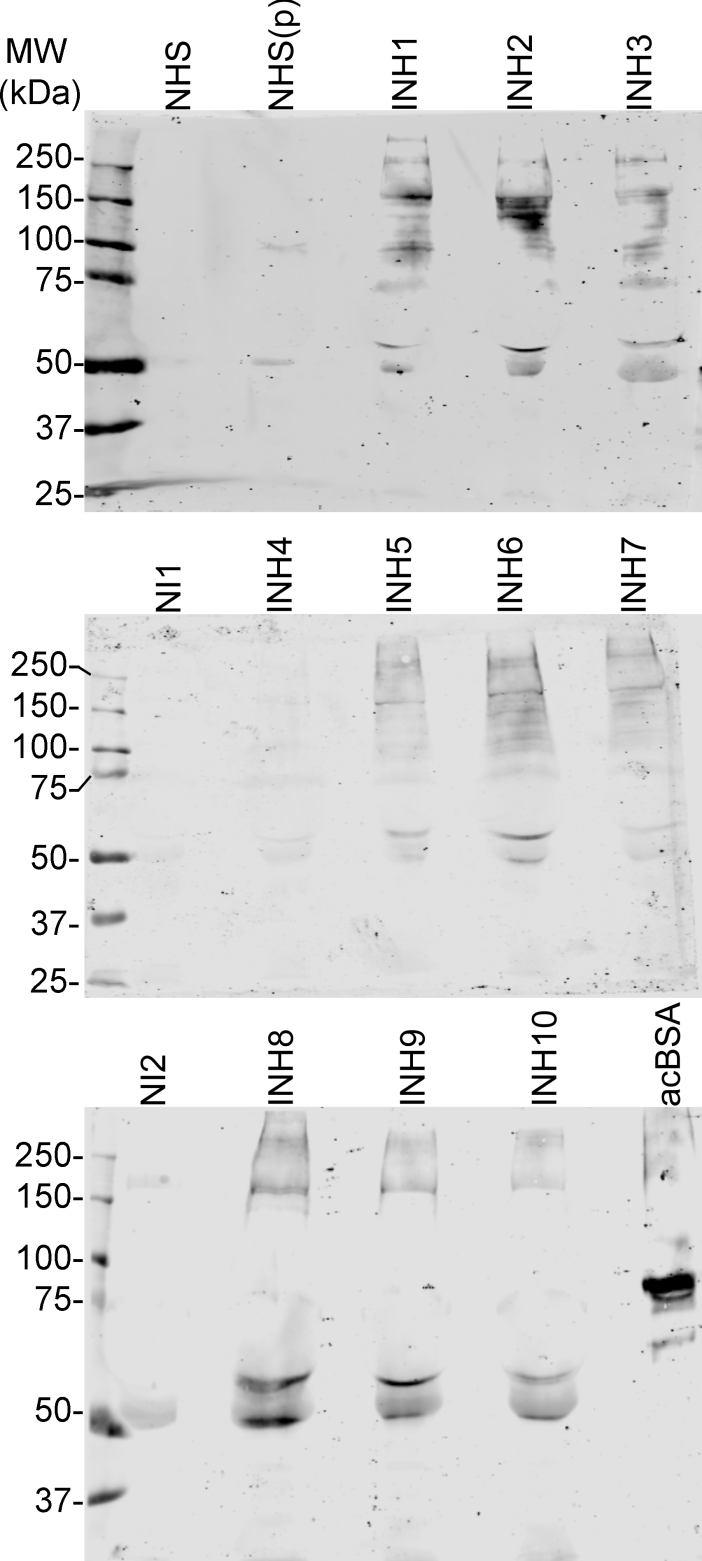
Inhibiting sera have ficolin-2-binding molecules. Normal human serum (NHS), NHS collected in a plastic tube [NHS(p)], noninhibiting archived sera (NI), inhibiting archived sera (INH), and acetylated BSA (acBSA). were assayed for ficolin-2-binding species as described in *Materials and Methods*. MW, molecular weight.

We hypothesized that the ficolin-2 inhibitors could be serum proteins with post-translational modifications to create ficolin-2 binding sites on proteins not normally recognized by ficolin-2. To visualize ficolin-2 ligands in serum, we performed far-western blotting on randomly-selected inhibiting and non-inhibiting serum samples. We also tested NHS collected in a plastic serum collection tube, which is known to introduce ficolin-2 inhibition (Brady et al., 2014c). While NHS exhibited little ficolin-2 reactivity whether collected in a glass or plastic tube (Fig. 6), inhibiting sera exhibited similar patterns of ficolin-2 reactivity between 100 kDa and 150 kDa.

## Discussion

A major feature of the aging process is inflammation (inflammaging), and increased complement levels may be associated with aging. For instance, CRP is elevated among older adults (Woloshin & Schwartz, 2005). Ficolin-2 binds a wide array of molecules and triggers the lectin pathway of complement activation, and thus it may be involved in inflammaging. However, our studies of archived sera from older persons show that ficolin-2 is not elevated, but many archived sera have ficolin-2 inhibitors that can interfere with a commercial ELISA and result in apparently low ficolin-2 levels. These inhibitors can be visualized by far-western blotting as distinct ficolin-2 ligands with apparent molecular weights between ∼100 and 150 kDa among sera from older adults. However, the inhibitors were absent in freshly collected serum from matched donors, and inhibitors were demonstrable in sera from younger persons after a long-term storage. Thus, the inhibitors were likely generated during storage and are not associated with aging.

The notion that the inhibitors appeared during storage was further supported by absence of correlation between ficolin-2 inhibition and various inflammaging parameters such as CRP and IL-6. Indeed, high IL-6 is a strong predictor of mortality among older adults (Harris et al., 1999), and neither group exhibited a significant difference in serum IL-6 or time to death. However, ficolin-2 inhibition was weakly correlated (*p* = 0.034) with the use of non-steroidal anti-inflammatory drugs (commonly known as NSAIDs). The patient population requiring the drugs may have appropriate serum factors that lead to creation of ficolin-2 inhibitors, or some drugs in the serum may predispose a sample to formation of ficolin-2 inhibitors during storage.

The molecular basis for ficolin-2 ligands in stored sera is unclear, but we may speculate concerning plausible theories for their origins. Serum proteins undergo changes during short and long-term storage; for example, antibodies can undergo chemical alteration via numerous processes, including oxidation and deamidation (Vlasak & Ionescu, 2008). Creatine kinase isoforms induced by serum carboxypeptidases appear within 4 h at 0 °C (George et al., 1984). In addition, many glycoproteins have N-linked glycosyl chains containing GlcNAc as an internal residue (Dall’Olio et al., 2013). Intact glycosyl chains may degrade during storage and may create a ficolin-2 ligand by exposing previously shielded GlcNAc moieties. This process may be more severe with older adults’ sera since agalactosylated N-linked glycans with exposed GlcNAc moieties are increased in individuals over 60 years old (Dall’Olio et al., 2013). Alternatively, individuals on long-term, high-dose aspirin therapy have lysine-acetylated serum proteins, including hemoglobin (Bridges et al., 1975) and albumin (Hawkins et al., 1969). Lysine-acetylation via aspirin can also occur *in vitro* (Hawkins, Pinckard & Farr, 1968); thus, certain NSAIDs (e.g., aspirin) may induce acetylation of proteins during storage. As serum storage at −80 °C has been known to preserve samples (Yang et al., 2015), it is difficult to envision an active process in the generation of ligands in serum stored at −80 °C. Nevertheless, cytokine levels have been reported to decrease after prolonged (>2 years) storage at −80 °C (De Jager et al., 2009).

Our current observation adds to the growing list of analytical factors that can influence assays for ficolin-2, an important innate opsonin. We and others have reported the specific inhibition of ficolin-2 by the coating in plastic serum collection tubes (Brady et al., 2014c; Hein et al., 2013), and others have observed the depletion of ficolin-2 by heparin-treated cardio bypass circuits (Hein et al., 2015). Ficolin-2 was observed to bind to derivatized Sepharose (Kilpatrick & Chalmers, 2012), and commercial complement component-depleted sera are deplete of ficolin-2 as well (Brady et al., 2014b). Ficolin-2 studies therefore require careful consideration of various analytical factors. The fact that ficolin-2 has proven to be a difficult molecule to study nevertheless supports the fact that ficolin-2 has broad binding specificity.

Infections by most pneumococcal capsule types are common in two extremes of age—namely, young children and older adults. Yet, infections by pneumococcal serotype 11A are common among among older adults (Pilishvili et al., 2010), especially those with chronic obstructive pulmonary disorder (Domenech et al., 2011), but are very rare among children (Brady et al., 2014a; Pilishvili et al., 2010). These epidemiologic findings suggest ficolin-2 provides protection in children (Brady et al., 2014a) but not older adults. As ficolin-2 levels appear normal in older adults, downstream elements of the ficolin-2 pathway may be dysfunctional. For instance, ficolin-2 requires MASPs to activate complement cascade and MASPs may be dysfunctional among elderly adults. In addition, phagocytes need to remove serotype 11A pneumococci after opsonization by ficolin-2 or complement. Neutrophils from older adults are less effective in phagocytosis (Simell et al., 2011), and macrophages decrease in function with age (Hearps et al., 2012). Thus, one needs to study the impact of aging on the entire pathway of ficolin-2-mediated protection using natural targets such as pneumococci.

There is increasing interest in the role of inflammation in aging, and complement activation is critical to inflammation. It is also clear that complement activation is complex, involving a large number of molecules in multiple activation pathways and regulatory steps. In addition to host defense, complement is critically important in other age-related ailments such as age-related macular degeneration (recently reviewed, (McHarg et al., 2015)), yet studies examining complement function among the aged are scarce and frequently contradictory ((Simell et al., 2011), and references therein). Given the wide range of biological activities mediated by complement, including the neutralization and removal of infectious agents and dead or dying cells, we must work to understand the changes to complement and its activators in the aging host and its implications for infectious diseases, autoimmune diseases, and inflammaging. Identifying the mechanism by which ficolin-2 may protect children but not older adults from serotype 11A pneumococcal infection may shed important light on these critical biological processes.

##  Supplemental Information

10.7717/peerj.2705/supp-1 Figure S1 Archived older adults’ sera are significantly reduced in ficolin-2 content relative to healthy young controls

10.7717/peerj.2705/supp-2 Figure S2 Some older individuals’ sera deposit disproportionately little ficolin-2 on serotype 11A bacteria

10.7717/peerj.2705/supp-3 Figure S3 Some older adults’ sera have heat-resistant ficolin-2 inhibition

10.7717/peerj.2705/supp-4 Figure S4A Inhibiting sera exhibit significantly reduced ficolin-2 by ELISA

10.7717/peerj.2705/supp-5 Figure S4B Inhibitors interfere with ficolin-2 quantitation by ELISA

10.7717/peerj.2705/supp-6 Figure S5 Freshly-collected sera from UAB Study of Aging participants whose archived samples inhibit ficolin-2 have significantly more ficolin-2 than their archived counterparts

## Additional Information and Declarations

### Data Availability

The following information was supplied regarding data availability: The raw data has been supplied as a Supplemental File.
